# Temporary heat stress suppresses PAMP‐triggered immunity and resistance to bacteria in *Arabidopsis thaliana*

**DOI:** 10.1111/mpp.12799

**Published:** 2019-03-29

**Authors:** Martin Janda, Lucie Lamparová, Alžběta Zubíková, Lenka Burketová, Jan Martinec, Zuzana Krčková

**Affiliations:** ^1^ University of Chemistry and Technology Prague Technická 5, Prague 6 – Dejvice 166 282 Czech Republic; ^2^ The Czech Academy of Sciences Institute of Experimental Botany Rozvojová 263, Prague 6 – Lysolaje 165 00 Czech Republic

**Keywords:** *Arabidopsis thaliana*, flagellin sensing 2 receptor, flg22, heat stress, PAMP‐triggered immunity, *Pseudomonas syringae*, reactive oxygen species

## Abstract

Recognition of pathogen‐associated molecular patterns (PAMPs) is crucial for plant defence against pathogen attack. The best characterized PAMP is flg22, a 22 amino acid conserved peptide from flagellin protein. In *Arabidopsis thaliana,* flg22 is recognized by the flagellin sensing 2 (FLS2) receptor. In this study, we focused on biotic stress responses triggered by flg22 after exposure to temporary heat stress (HS). It is important to study the reactions of plants to multiple stress conditions because plants are often exposed simultaneously to a combination of both abiotic and biotic stresses. Transient early production of reactive oxygen species (ROS) is a well‐characterized response to PAMP recognition. We demonstrate the strong reduction of flg22‐induced ROS production in *A. thaliana* after HS treatment. In addition, a decrease in *FLS2 *transcription and a decrease of the FLS2 presence at the plasma membrane are shown after HS. In summary, our data show the strong inhibitory effect of HS on flg22‐triggered events in *A. thaliana*. Subsequently, temporary HS strongly decreases the resistance of *A. thaliana* to *Pseudomonas syringae*. We propose that short exposure to high temperature is a crucial abiotic stress factor that suppresses PAMP‐triggered immunity, which subsequently leads to the higher susceptibility of plants to pathogens.

Plants are exposed to different environmental stresses that often occur simultaneously. Due to their sessile disposition, plants had to develop strategies to adapt to multi‐stress conditions. Nowadays, as the case of global warming becomes a very important issue, the studies of how high temperatures impact on plant resistance to pathogens are of the highest interest. Indeed, it is not exceptional now that even in the relatively mild climate areas temperature rises up to 40 °C or that the night and day temperature difference exceeds a variance of about 30 °C. This unstable temperature influences plant‐pathogen interactions amongst other stresses (Hua, 2013; Velásquez *et al*., 2018).

A typical response to pathogen attack is the activation of plant immunity. It consists of two layers: PTI (PAMP‐triggered immunity) and ETI (effector‐triggered immunity) (Jones and Dangl, 2006). PTI involves the recognition of conserved pathogen‐associated molecular patterns (PAMPs) acting as elicitors. Typical PAMPs include flagellin, peptidoglycan, Tu elongation factor, lipopolysaccharides, chitin and glucans. PAMPs are recognized at the plasma membrane (PM) by specific pattern recognition receptors (PRRs) (Boller and Felix, 2009; Trda *et al*., 2015). The best characterized plant PRR is flagellin sensing 2 (FLS2) that recognizes a conserved 22 amino acid peptide (flg22) from bacterial flagellin (Gomez‐Gomez *et al*., 1999; Zipfel *et al*., 2004). Flg22 recognition leads to the production of reactive oxygen species (ROS), nitric oxide, ion fluxes, cytoskeletal remodelling, stomatal closure, activation of protein phosphorylation cascades and increased levels of phytohormones (salicylic acid [SA], jasmonic acid [JA] and ethylene) (Bigeard *et al*., 2015). Subsequently, the transcription of defence genes is induced, followed by the expression of antimicrobial compounds and the strengthening of the cell wall by the accumulation of callose (Boller and Felix, 2009; Muthamilarasan and Prasad, 2013). Pathogenic organisms have developed special molecules, called effectors, to overcome PTI. Effectors are recognized by the receptor proteins (mostly intracellular) commonly called R‐proteins. This recognition leads to ETI responses that are similar to PTI, but are often stronger and could result in the hypersensitive reaction (HR) (Chisholm *et al*., 2006; Coll *et al*., 2011; Cui *et al*., 2015; He *et al*., 2006). In this study, we focused on the effect of temporary heat stress (HS) on *Arabidopsis thaliana* PTI and the resistance to *Pseudomonas syringae* pv. *tomato* (*Pst*) DC3000.

Several studies focused on the relationship between HS and plant immunity. However, until now most studies used ‘long‐term’ exposure to HS in their experiments, such as 1 day or constitutive growth under mildly elevated temperature (approximately 28 °C) (Cheng *et al*., 2013; Wang *et al*., 2009; Zhu *et al*., 2010). Based on a gene transcription and mitogen‐activated protein kinase (MAPK) activation analysis, it was shown that PTI is activated within a temperature interval of 23 °C–32 °C with the peak at 28 °C (Cheng *et al*., 2013). We were then interested in situations, while plants suffered more severe HS than a mildly elevated temperature. In this study, we show that short exposure to high temperature suppressed typical PTI responses. The ROS production, one of the best characterized responses to pathogen attack, increased after exposure to flg22 (Smith and Heese, 2014), as well as after HS (Volkov *et al*., 2006; Wang *et al*., 2014). We observed that the exposure of 4‐week‐old *A. thaliana* leaf discs to 42 °C for 45 min almost blocks the transient flg22‐induced ROS production, which normally occurs within approximately 12 min (Methods S1, Fig. 1A). The exposure of the leaf discs to HS alone increased ROS production as late as in 40 min after exposure to 42 °C (Fig. 1A inset). It confirms a distinct origin of ROS in the response of plant immunity and in reaction to HS. The HS‐induced inhibition of flg22‐induced ROS production is time‐dependent; 15 min at 42 °C was not enough to suppress ROS production, but when the leaf discs were exposed to HS for 30 min, the maximum ROS value was reduced to 13% and even nearly blocked after 45 min exposure (Fig. 1B). The reduction of the flg22‐induced ROS production after HS might be the consequence of damage to plants by applied HS. Therefore, we tested whether the reduction is reversible. Leaf discs were returned after exposure to HS (45 min at 42 °C) to the control conditions (22 °C) for either 1 h or 2 h, and flg22‐induced ROS production was then monitored. In this case, ROS production did not fully recover (Fig. 1C). However, it was clear, and the recovery reached over 30% of the control. This suggests that HS did not cause any pathological or devastating changes to the plants under our experimental conditions. In addition, we found the same trend for lower temperatures, but a longer exposure time was needed. Flg22‐induced ROS production was severely decreased when the leaf discs were maintained at 37 °C for 120 min. After 360 min at 32 °C or 28 °C, the ROS production decreased to 14% or 65%, respectively (Figs 1D and S1). To address the specificity of the studied phenomenon, we tested another well‐known PTI‐triggering elicitor, a peptide from the N‐terminus of EF‐Tu (elf18). Elf18‐triggered responses are similar to the flg22‐triggered ones (Henty‐Ridilla *et al*., 2014; Kunze *et al*., 2004). In this study, HS induced the inhibition of elf18‐induced ROS production similarly to that of flg22. The elf18‐induced ROS production was reduced to 4% after 45 min at 42 °C, and after 2 h, the recovery reached 36% of the control and to 8% after 120 min at 37 °C, and after 2 h, the recovery reached 33% of the control (Fig. S2). We show that temporary HS has a robust inhibitory effect on PTI‐induced ROS production.

**Figure 1:**
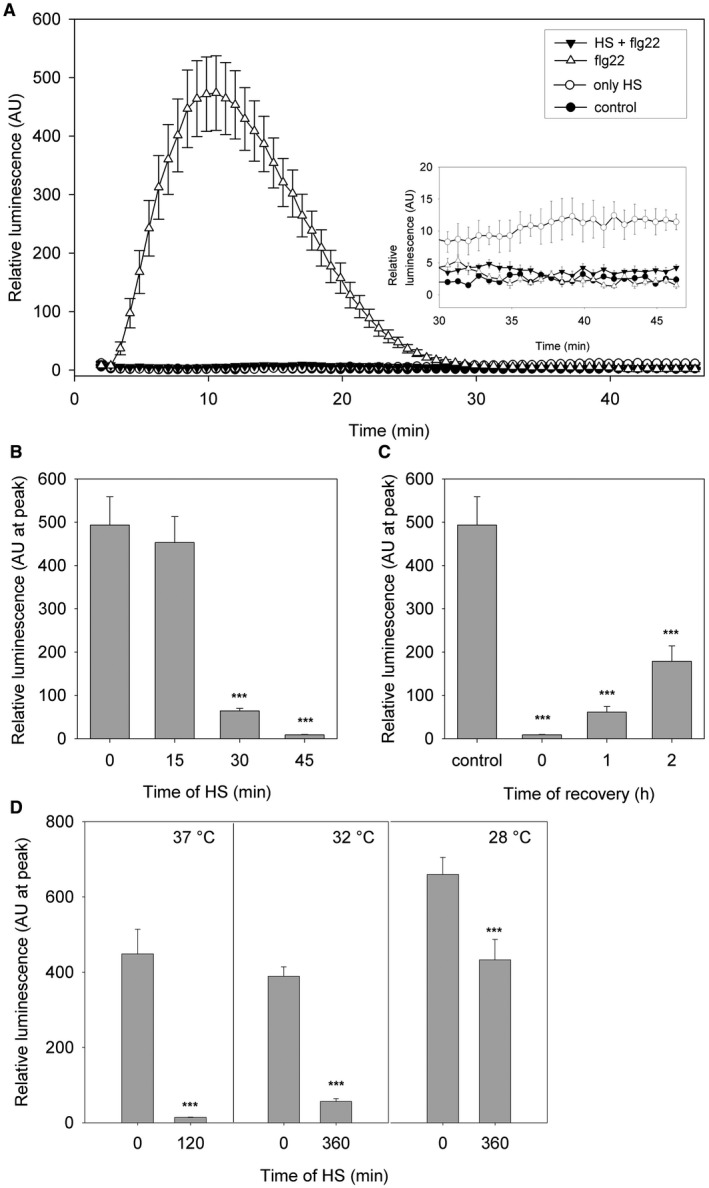
Oxidative burst triggered by the combination of HS and 100 nM flg22. The ROS production was measured by luminol‐dependent chemiluminescence counting in leaf discs from 4‐week‐old *Arabidopsis thaliana.* A) The ROS production kinetics monitored over 45 min after 100 nM flg22 (white triangle), after HS (42 °C at 45 min; white circle), after HS with a follow‐up treatment with flg22 (black triangle) and in control conditions (black circle). B) ROS production (represented by the peak luminescence) was measured after the addition of 100 nM flg22. The discs were pre‐treated with HS (15 min, 30 min and 45 min at 42 °C) or kept in control conditions (0 h). C) Recovery of the ROS production (represented by the peak luminescence) was measured after the addition of 100 nM flg22. The discs were pre‐treated with HS (45 min at 42 °C) or kept in control conditions. ROS production was measured immediately after HS (0 h), or the discs were returned to the control conditions for 1 h or 2 h and then measured. D) ROS production (represented by the peak luminescence) was measured after the addition of 100 nM flg22. The discs were pre‐treated with HS (120 min at 37 °C, 360 min at 32 °C and 360 min at 28 °C) or kept in control conditions (0 h). The data represent the means + SE; *n* = 8 leaf discs in one biological experiment. The experiment was repeated three times independently with similar results. Asterisks indicate that the mean value is significantly different from the control conditions without HS (two‐tailed Student's *t*‐test, *n* = 8, ***P < 0.001). HS, heat stress; ROS, reactive oxygen species; SE, standard error.

Flg22‐triggered ROS production starts with the recognition of flg22 by its plasma membrane‐localized receptor FLS2. In its non‐activated state, FLS2 constitutively cycles between the PM and early endosome compartments (Beck *et al*., 2012; Robatzek *et al*., 2006; Zipfel *et al*., 2004). Thus, the HS‐induced decrease of flg22‐triggered ROS production might be caused by the decrease of FLS2 transcription or by altered FLS2 cycling. We observed that the transcription level of *FLS2* decreased after 30 min and 45 min at 42 °C and after 120 min at 37 °C (Fig. 2A, Methods S1, Table S3). Transcriptional levels increased back to the original level after 2 h at 22 °C after HS (Fig. 2B). These results demonstrate the transient effect of temporary exposure to high temperature on *FLS2* transcription. To study the effect of HS on FLS2 protein level, we used plants harbouring *pFLS2::FLS2‐GFP‐HA *(Beck *et al*., 2012). A Western blot analysis of the PM fractions isolated from control and heat stressed plants (42 °C at 1 h) show a decreased level of FLS2 at the PM after HS (Fig. 2C). We used plants harbouring *p35S::FLOT2:GFP*, another PM protein (Daněk *et al*., 2016; Junková *et al*., 2018) to figure out whether the decrease of protein association with PM is a general effect of HS. In plants exposed to HS (1 h at 42 °C) FLOT2‐GFP level was not decreased in comparison to control conditions (Fig. S3). The specific decrease of FLS2 at PM after HS implies a mechanism by which HS suppresses flagellin‐activated responses. We suggest that the HS inhibited the *FLS2* transcription and decreased the amount of FLS2 at the PM, which results in the inhibition of flg22‐induced ROS production. This decrease of *FLS2* transcription after HS was transient, as well as the flg22‐induced ROS production (Figs 1 and 2).

**Figure 2:**
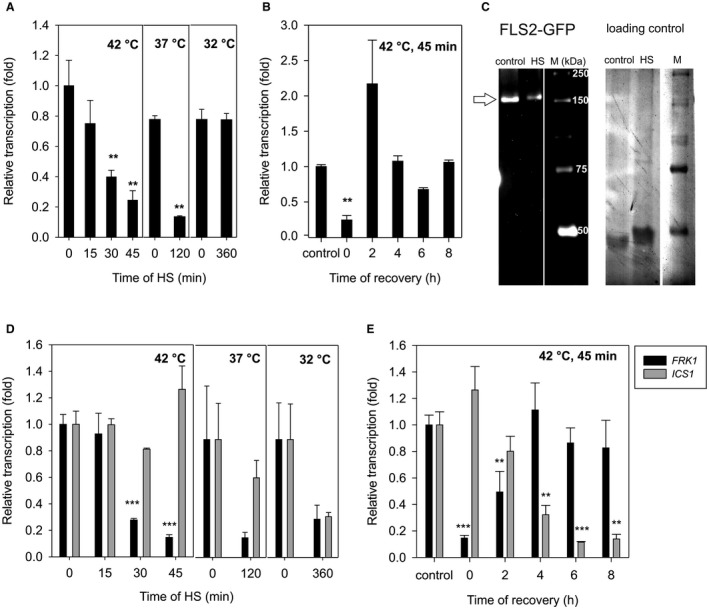
Analysis of *FLS2*, *FRK1* and *ICS1* transcription and the level of FLS2 at the PM after HS. Discs of 4‐week‐old *Arabidopsis thaliana* leaves were subjected to HS and transcript levels of the *FLS2* (A, B), *FRK1* and *ICS1* (D, E) were measured by quantitative real‐time PCR. (A, D) Leaf discs were stressed for 15 min, 30 min and 45 min at 42 °C; 120 min at 37 °C and 360 min at 32 °C. (B, E) Leaf discs were stressed for 45 min at 42 °C and were returned to the control conditions for 2 h, 4 h, 6 h and 8 h. *TIP41* was used as a reference gene. The data represent the means + SE; *n* = 3 discrete samples from one biological experiment. The experiment was repeated three times independently with similar results. Asterisks indicate that the mean value is significantly different from the control conditions (two‐tailed Student's *t*‐test, *n* = 3, **P < 0.01, ***P < 0.001). (C) Western blot analysis of the PM was performed. The PM was purified from a 4‐week‐old leaf of *A. thaliana* plants harbouring pFLS2::FLS2‐GFP‐HA. These plants were treated with either HS (42 °C at 1 h) or kept in control conditions. The GFP tag was detected using anti‐GFP antibody. Loading controls of proteins on membrane were visualized using Novex reversible membrane protein stain. HS, heat stress; FLS, flagellin‐sensitive2; FRK1, FLG22‐induced receptor‐like kinase1; GFP, green fluorescent protein; ICS1, isochromate synthase 1; M, marker; PCR, polymerase chain reaction; PM, plasma membrane; SE, standard error.

Furthermore, we wondered whether other PTI‐induced genes were inhibited by the temporary HS. We analysed the transcription of two genes (*FRK1* and *ICS1*) activated after the recognition of flg22 (Swain *et al*., 2015; Tsuda *et al*., 2008; Wildermuth *et al*., 2001; Yeh *et al*., 2015). It was reported that *FRK1 *transcription is activated early upon the recognition of the PAMPs (including flg22), and it its transcription is enhanced by temperatures between 25 °C–32 °C during the response to flg22 (Cheng *et al*., 2013). However, the authors used *A. thaliana* protoplasts exposed to 32 °C for just 15 min, 3 h before flg22 treatment. In our experiments, *FRK1* transcription was transiently but inhibited by the exposure to 42 °C for 45 min (Fig. 2D,E). This inconsistency indicates a very important role of timing and/or difference between basal and elicited responses of immune components upon HS. *ICS1* is one of the crucial components of the SA biosynthetic pathway (Wildermuth *et al*., 2001). *ICS1* transcription induced by flg22 (or *Pst* DC3000) is strongly inhibited at 30  C followed by a decrease of SA concentration (Huot *et al*., 2017). In our conditions, the transcription of *ICS1* decreased at a later time during the recovery phase after HS (42 °C at 45 min) (Fig. 2E). This is consistent with the slower transcriptional reaction of this gene in comparison to *FRK1* (Denoux *et al*., 2008; Hunter *et al*., 2013). To study whether the PTI‐activated gene transcription response is generally suppressed after HS, we performed *in silico* gene transcription analysis using Genevestigator (Hruz *et al*., 2008). We selected 20 genes connected with plant immunity in general and with PTI in particular (Table S1) (Muthamilarasan and Prasad, 2013). Genes specifically connected to PTI include *FLS2*, *BAK1* and *BIK1*. PTI overlaps with ETI signalling with the activation of such genes as those involved in the *MAPK* cascade (*MKK4*, *MEKK1*, *MKK1* and *MPK4*) and *WRKY* transcription factors (Pandey and Somssich, 2009). EDS1, PAD4 and SAG101 proteins that mediate the SA‐signalling result in *PR* (pathogenesis related) and *NPR* (nonexpressor of pathogenesis‐related genes 1) induced transcription (Makandar *et al*., 2015). Using Genevestigator, we show that those genes have higher transcription after differential treatment with flg22 (Fig. S4). However, the transcription of these genes was largely inhibited in the experiments where the plants were exposed to high temperature (Fig. S4). Figure S4 also shows an example of genes activated by HS (*HSFA7A*, *HSA32*, *HSP18* and *HSP70*). Our analysis is consistent with that of Huot *et al. *(2017) who showed that after benzothiadiazole (a functional analogue of SA) treatment, a high proportion of the induced genes was suppressed at 30 °C, including *PR1, PR2, ICS1, SARD1, EDS1, PAD4, WRKY18 *and *WRKY60*. Overall, the transcription of the flg22‐activated genes is inhibited by temporary HS.

In addition to ROS production and the transcription of defence‐related genes, the PTI response is characterized by callose deposition. Zhang *et al*. (2007) showed that respiratory burst oxidase homolog D (RbohD) regulates cell wall defence exemplified by callose deposition. Callose deposition is influenced by the SA‐signalling pathway (Huot *et al*., 2017; Yi *et al*., 2014). We performed our experiments on leaf discs from 4‐week‐old plants as used for the ROS measurement. We induced callose formation by flg22 treatment in the leaf discs. We observed that the callose formation decreased to 60% when the leaf discs were exposed to HS (42 °C for 1 h) and treated with flg22 at 22 °C for 24 h compared to the control conditions at 22 °C (Fig. S5). Consistently with the results from Huot *et al*. (2017), callose accumulation was almost blocked when the treatment with flg22 was performed under the heat conditions (37 °C; Fig. S5), but in this case, the leaf discs were constantly subjected to high temperature (24 h).

To summarize, our results show that temporary HS caused the inhibition of the early flg22‐triggered responses of plant immunity (ROS production and *FLS2* transcription), as well as later responses (transcriptional activation of genes of plant immunity pathways and callose deposition).

As a final point, we wanted to determine if temporary HS could also affect the resistance of *A. thaliana* to *Pst* DC3000. The inhibitory effect of longer exposure (24 h or more) to mildly elevated temperature on plant immunity, especially on the SA‐signalling pathway, had been shown. Mutants with elevated SA production, such as *bon1*, *cpr1* and *snc1, *are very good examples. The dwarf phenotype of these mutants and SA production was suppressed during constant growth at 28 °C (Bowling *et al*., 1994; Felix *et al*., 1999; Hammoudi *et al*., 2018; Ichimura *et al*., 2006; Janda and Ruelland, 2015; Yang and Hua, 2004; Zhang *et al*., 2003). The exposure to mildly elevated temperature (28 °C–30 °C) also inhibits *A. thaliana* resistance to *Pst* DC3000 (Huot *et al*., 2017; Menna *et al*., 2015; Wang *et al*., 2009; Xin *et al*., 2018). It must be noted that in those studies, the plants were exposed to elevated temperature also after the treatment with *Pst* DC3000. Therefore, it is difficult to conclude that the higher susceptibility is caused solely by HS because the bacteria were also affected by elevated temperature.

In this study, we used a higher temperature for the temporary exposure of plants (1 h at 42 °C and 2 h at 37 °C) before bacterial treatment. After HS, the plants were kept in control conditions (22 °C at 18 h), subsequently infiltrated with *Pst *DC3000 and maintained at 22 °C. As a result, in our experimental design *Pst* DC3000 propagation was not affected by the high temperature and all the observed effects on *Pst *DC3000 growth *in planta* came from the changes in host metabolism. To overcome the immunity connected with stomata closure, which could be affected by higher temperature, we infiltrated *Pst* DC3000 directly into the apoplast. Indeed, we demonstrated that *A. thaliana* plants exposed to 42 °C or 37 °C were more susceptible to *Pst *DC3000 comparing to the control (Fig. 3A). The bacterial growth in plants at 42 °C and 37 °C was fourfold higher than at 22 °C.

**Figure 3:**
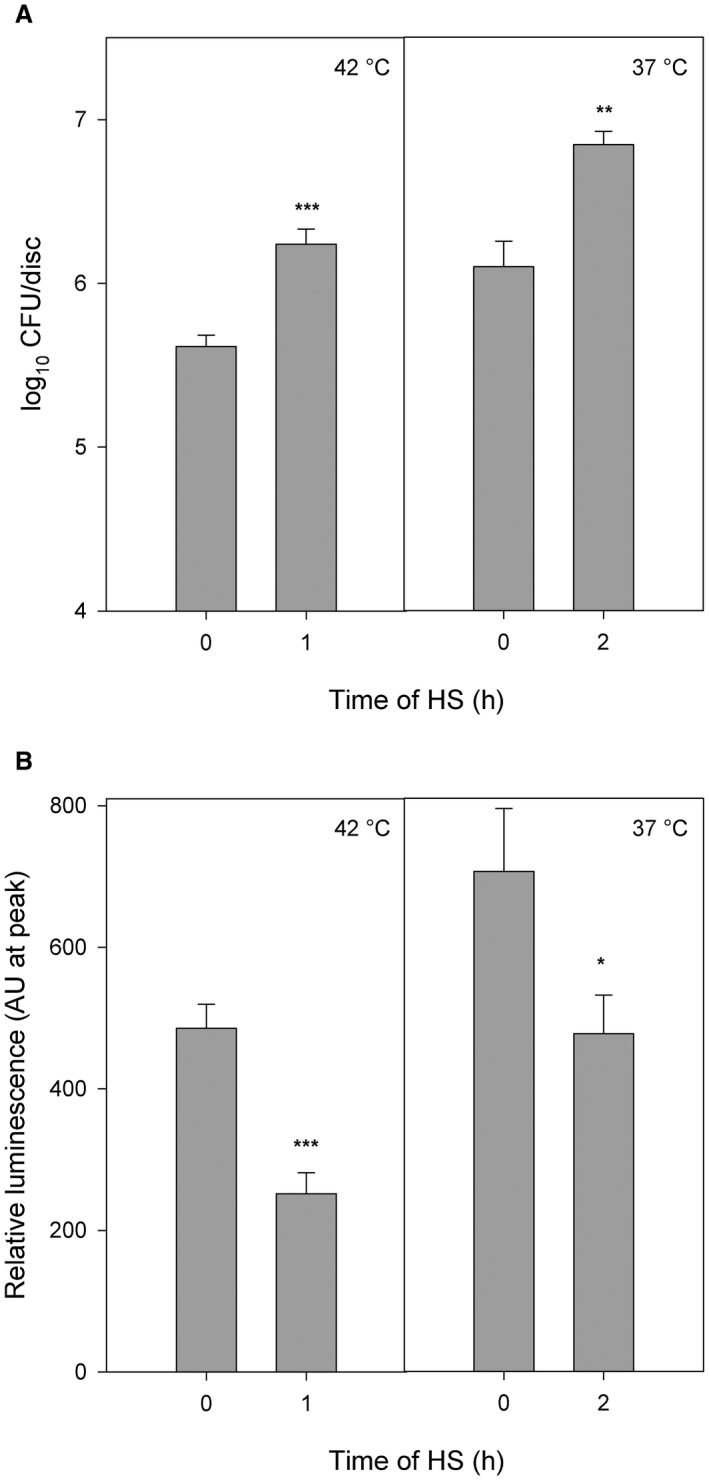
Resistance to *Pst* DC3000 and the oxidative burst triggered by flg22 after temporary HS. (A) Four‐week‐old *Arabidopsis thaliana* plants were kept at 42 °C for 1 h and at 37 °C for 2 h. The recovery phase took 18 h in control conditions before treatment with *Pst* DC3000. Control plants were continuously cultivated at 22 °C. Leaf discs were harvested 3 days after inoculation with *Pst* DC3000. The data represent the means + SE; *n* = 4 from four biological experiments. (B) The plants were exposed to 42 °C for 1 h and 37 °C for 2 h. After HS, the leaf discs were cut and returned to 22 °C for 18 h. ROS production (represented by the peak luminescence) was measured after the addition of 100 nM flg22. The data represent means + SE; *n* = 12 discs. The experiment was repeated three times independently with similar results. The asterisks represent statistically significant changes between the heat and control conditions (*P < 0.5, **P < 0.01, ***P < 0.001; two‐tailed Student´s *t*‐test). CFU, colony‐forming units; HS, heat stress; *Pst*, Pseudomonas syringae pv. tomato, ROS, reactive oxygen species; SE, standard error.

To determine if the effect of a long recovery from HS on resistance exists at the level of ROS production, we treated whole plants with HS (1 h at 42 °C; 2 h at 37 °C), cut the leaf discs and left them at 22 °C for 18 h. In this case, the ROS production was decreased to 50% at the plants treated with 42 °C and to 68% at the plants treated with 37 °C (Fig. 3B). It means that temporary HS led to the significant inhibition of flg22‐induced ROS production.

To address whether full recovery after HS is possible, we returned plants after exposure to HS (2 h at 37 °C or 1h at 42 °C) to the control conditions (22 °C) for 24 h, cut the leaf discs and left them at 22 °C for 18 h. Indeed, we were able to detect the full recovery for the 37 °C treated plants and 70% recovery for the plants exposed to 42 °C (Fig. S6). We wondered whether the general physiological parameters were affected in our HS conditions. To study this, we measured ion leakage at 24 h after HS using a COND70 conductivity meter (XS Instruments) (Prerostova *et al*., 2018) and detected no difference between control and HS‐treated plants (Table S2). In addition 6 days after the HS exposure (1 h at 42 °C) plants still looked like control plants (Fig. S7). This indicates that used mild HS conditions did not cause severe damage on plants.

It is possible, that the inhibition of PTI is one of the reasons why plants are vulnerable to pathogen attack even 18 h after the exposure to high temperature. Huot *et al*. (2017) showed decreased resistance of *A. thaliana* after *Pst* DC3000 infection, but only when the plants were maintained at 30 °C. However, when plants were exposed to 30 °C for 2 days before infiltration and subsequently subjected to 23 °C, there was no detectable difference in the infection development comparing to the control plants. It might seem that just temporary mild HS is not sufficient to affect resistance. Alternatively, here we show that temporary effect of higher temperatures (37 °C and 42 °C) decreased the resistance of *A. thaliana* to *Pst* DC3000 without the influence of temperature on the pathogen.

In conclusion, here we provide the evidence that the modulation of plant metabolism by HS leads to higher susceptibility to the bacterial pathogen (Fig. 3). We present a broad study of flg22‐triggered ROS production upon short exposure to high temperatures, which demonstrated a strong inhibitory effect of temporary HS on ROS production. In parallel, the transcription of the genes activated by flg22 was inhibited after HS. We suggest that these processes play an important role in the decreased amount of FLS2 at the PM after HS. From those results, it could be stated that HS significantly influences the PTI and resistance to *Pst* DC3000 in *A. thaliana*.

## Accession numbers

FLS2, At5g46330; FRK1, At2g19190; ICS1, At1g74710; and TIP41, At4g34270.

## Supporting information


**Fig. S1** Oxidative burst triggered by the combination of HS and 100 nM flg22. The ROS production was measured by luminol‐dependent chemiluminescence counting in leaf discs from 4‐week‐old *A. thaliana*. ROS production (represented by the peak luminescence) was measured after the addition of 100 nM flg22. The discs were pre‐treated with HS (90 min and 120 min at 37 °C, 60; 120 min and 360 min at 32 °C; and 60 min, 120 min and 360 min at 28 °C) or kept in control conditions (0 h). The data represent the means  +SE; *n* = 8 leaf discs in one biological experiment. The experiment was repeated two times independently with similar results. Asterisks indicate that the mean value is significantly different from the control conditions without HS (two‐tailed Student's *t*‐test, *n* = 8, *P < 0.05, ***P < 0.001). HS, heat stress; ROS, reactive oxygen species; SE, standard error.

**Fig. S2** Oxidative burst triggered by the combination of HS and 100 nM elf18. The ROS production was measured using luminol‐dependent chemiluminescence counting in leaf discs from 4‐week‐old *A. thaliana*. ROS production (represented by the peak luminescence) was measured after the addition of 100 nM elf18. The discs were pre‐treated with HS (45 min at 42 °C or 120 min at 37 °C) or kept in control conditions. The production of ROS was measurement immediately after HS (0 h), or the discs were returned to the control conditions for 1 h or 2 h. The data represent the means + SE; *n* = 8 leaf discs in one biological experiment. The experiment was repeated twice independently with similar results. Asterisks indicate that the mean value is significantly different from the control conditions without HS (two‐tailed Student's *t*‐test, *n* = 8, **P < 0.01, ***P < 0.001). HS, heat stress; ROS, reactive oxygen species, SE, standard error.

**Fig. S3** The level of FLOT2 at the PM after HS. Western blot analysis of the PM was performed. The PM was purified from leaf of 4‐week‐old *A. thaliana* plants harbouring p35S::Flot2:GFP. Amount of protein was 2.7 μg per lane. These plants were treated with either HS (42 °C for 1 h) or kept in control conditions. Immunoblotting analysis of FLOT2 with GFP tag was detected using anti‐GFP antibody. Loading controls of proteins on membrane were visualized using Novex reversible membrane protein stain. HS, heat stress; FLOT2, flotillin 2; GFP, green fluorescent protein; PM, plasma membrane.

**Fig. S4** *In silico* analysis of the transcription of genes involved in plant immunity and HS. Transcription pattern of plant immunity response genes in *A. thaliana*. Diagrams show that selected genes responded differently to treatment by flg22 and HS. Experimental data were performed using Genevestigator (http://www.genevestigator.com). For list of genes see Table S1. HS, heat stress.

**Fig. S5** Callose deposition triggered by the combination of HS and flg22. Callose was stained with aniline blue and observed using fluorescent microscopy. The graph displays the amount of callose spots per 1 disc from 4‐week old *A. thaliana* plants treated with flg22 (100 nM, grey box) or without flg22 (black box) and with HS, A) 1 h at 42 °C and B) 2 h at 37 °C; 24 h at 37 °C. Treatment with flg22 took 24 h, and HS was applied either 1 h before the addition of flg22 (in the case of 1 h at 42 °C or 2 h at 37 °C treatment) or simultaneously with the flg22 treatment (in the case of 24 h at 37 °C). The data represent the means + SD; *n* = 30 leaf discs. One biological experiment was comprised of ten leaf discs. Values with different letters differed significantly at *P* < 0.05 based on a one‐way analysis of variance (ANOVA) with a post‐hoc Tukey HSD test. HS, heat stress, SD, standard deviation.

**Fig. S6** Oxidative burst triggered by flg22 after temporary HS. Four‐week‐old *A. thaliana* plants were exposed to 42 °C for 1 h and 37 °C for 2 h. After HS the plants were put back to the control conditions for 24 h. After that the leaf discs were cut and returned to 22 °C for 18 h. ROS production (represented by the peak luminescence) was measured after the addition of 100 nM flg22. The data represent means + SE; *n* = 12 discs. The experiment was repeated three times independently with similar results. The asterisks represent statistically significant changes between the heat and control conditions (*P < 0.5; two‐tailed Student´s *t*‐test). HS, heat stress; ROS, reactive oxygen species, SE, standard error.

**Fig. S7** The phenotype of *A. thaliana* after heat stress (HS). Four‐week‐old *A. thaliana* plants were kept at 42 °C for 1 h and put back to the control conditions; 6 days after HS the pictures were taken.

**Table S1** The phenotype of *A. thaliana* after heat stress (HS). Four‐week‐old *A. thaliana* plants were kept at 42 °C for 1 h and put back to the control conditions; 6 days after HS the pictures were taken.

**Table S2** Ion leakage 24 h after heat stress (HS) measured with a conductivity metre.

**Table S3** Sequence of specific primers.

**Methods S1** Experimental procedures.
