# Supplementary figures and images for: Temporary heat stress suppresses PAMP‐triggered immunity and resistance to bacteria in Arabidopsis thaliana
Source: Mol Plant Pathol. 2019 Mar 29;20(7):1005–12. doi: 10.1111/mpp.12799 (PMC6589723; doi:10.1111/mpp.12799)

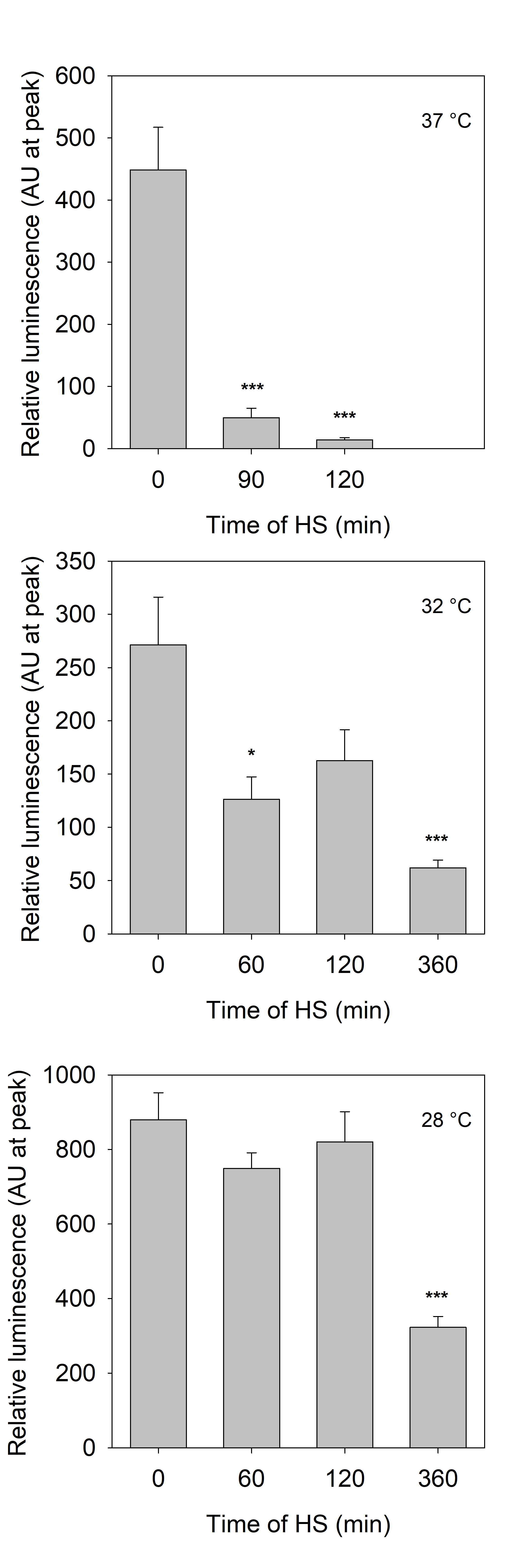

Supplement: Supplementary file 1 — Fig. S1 Oxidative burst triggered by the combination of HS and 100 nM flg22. The ROS production was measured by luminol‐dependent chemiluminescence counting in leaf discs from 4‐week‐old A. thaliana. ROS production (represented by the peak luminescence) was measured after the addition of 100 nM flg22. The discs were pre‐treated with HS (90 min and 120 min at 37 °C, 60; 120 min and 360 min at 32 °C; and 60 min, 120 min and 360 min at 28 °C) or kept in control conditions (0 h). The data represent the means +SE; n = 8 leaf discs in one biological experiment. The experiment was repeated two times independently with similar results. Asterisks indicate that the mean value is significantly different from the control conditions without HS (two‐tailed Student's t‐test, n = 8, *P < 0.05, ***P < 0.001). HS, heat stress; ROS, reactive oxygen species; SE, standard error. [file MPP-20-1005-s001.JPG]

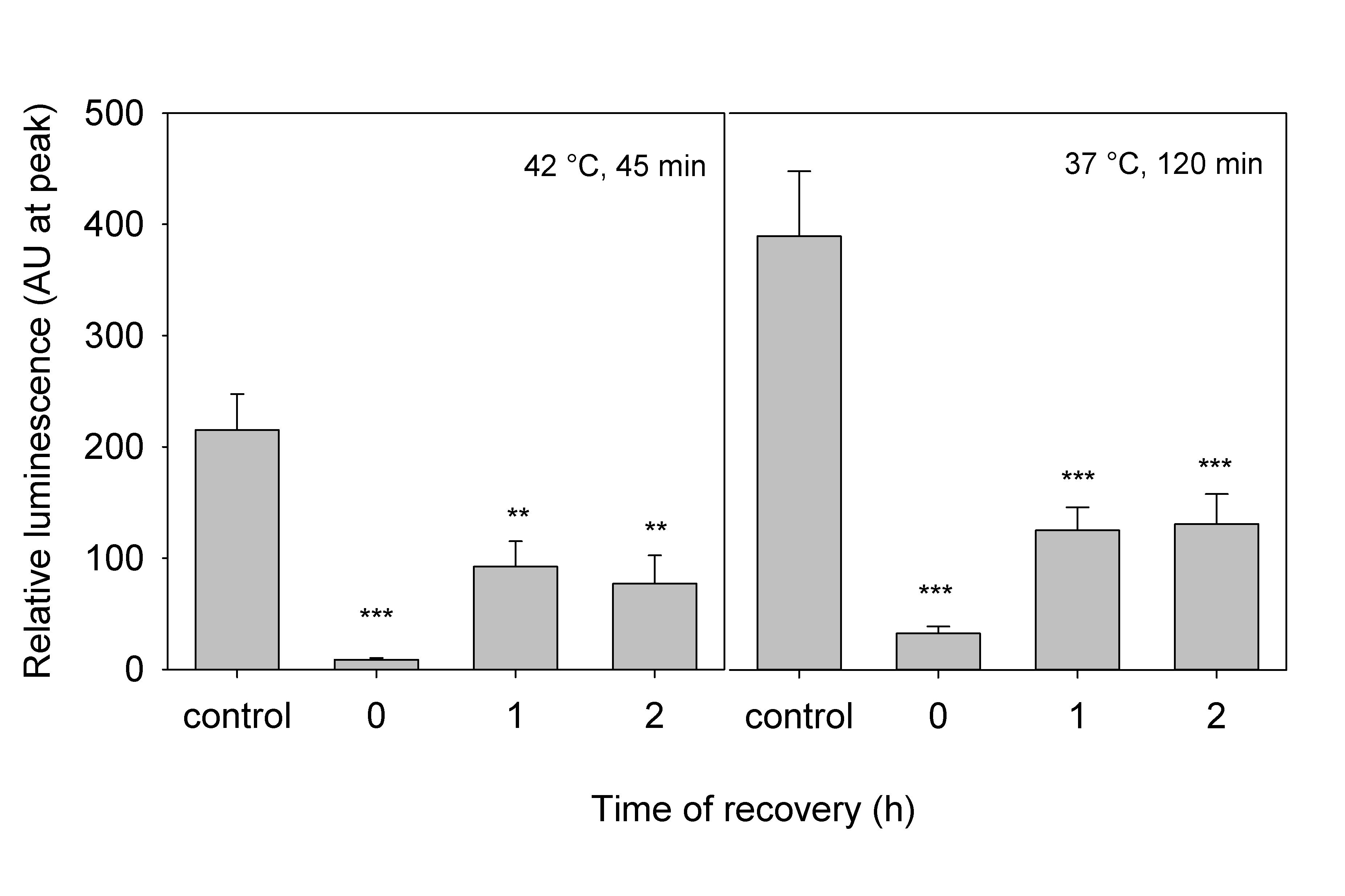

Supplement: Supplementary file 2 — Fig. S2 Oxidative burst triggered by the combination of HS and 100 nM elf18. The ROS production was measured using luminol‐dependent chemiluminescence counting in leaf discs from 4‐week‐old A. thaliana. ROS production (represented by the peak luminescence) was measured after the addition of 100 nM elf18. The discs were pre‐treated with HS (45 min at 42 °C or 120 min at 37 °C) or kept in control conditions. The production of ROS was measurement immediately after HS (0 h), or the discs were returned to the control conditions for 1 h or 2 h. The data represent the means + SE; n = 8 leaf discs in one biological experiment. The experiment was repeated twice independently with similar results. Asterisks indicate that the mean value is significantly different from the control conditions without HS (two‐tailed Student's t‐test, n = 8, **P < 0.01, ***P < 0.001). HS, heat stress; ROS, reactive oxygen species, SE, standard error. [file MPP-20-1005-s002.JPG]

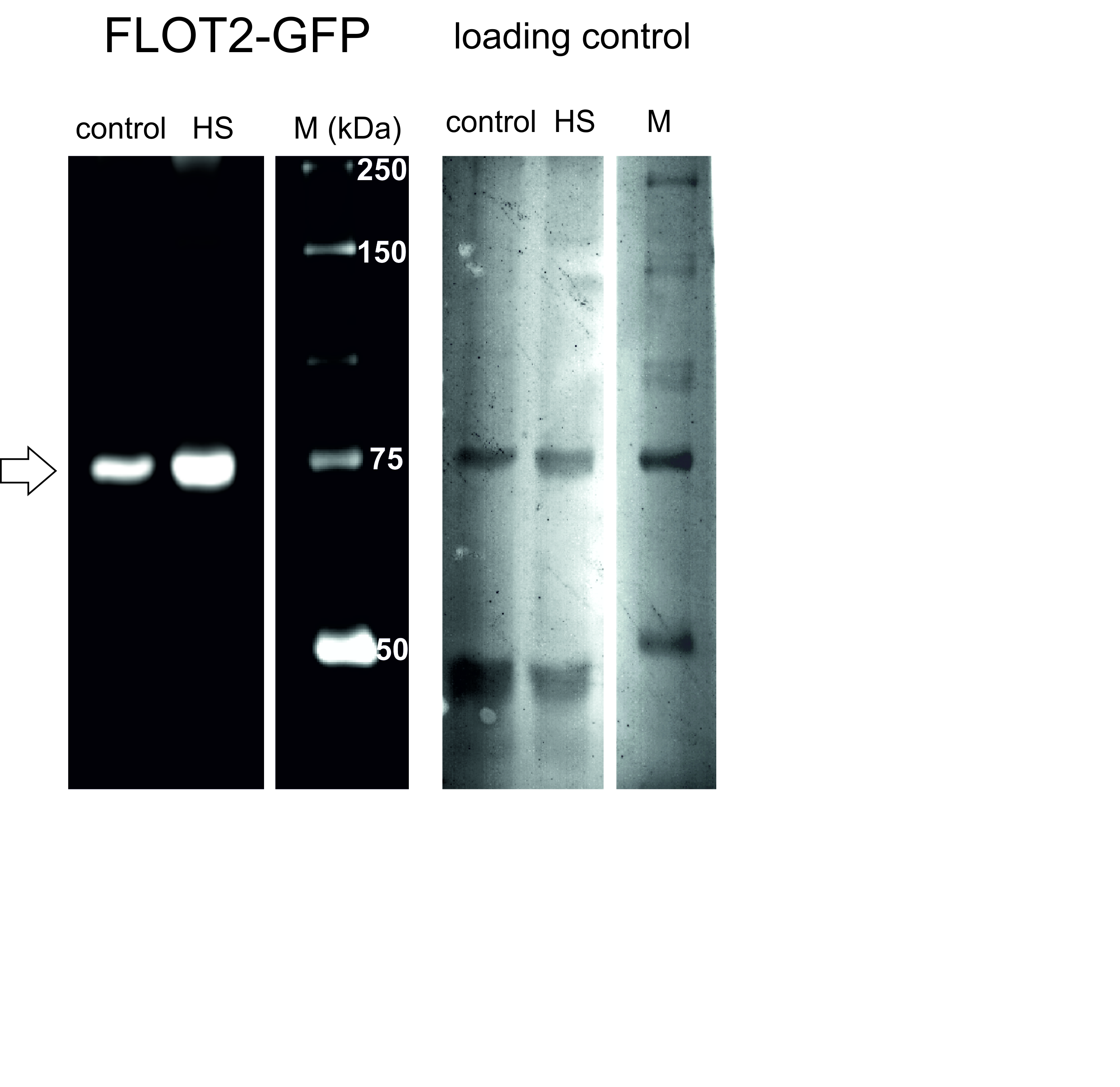

Supplement: Supplementary file 3 — Fig. S3 The level of FLOT2 at the PM after HS. Western blot analysis of the PM was performed. The PM was purified from leaf of 4‐week‐old A. thaliana plants harbouring p35S::Flot2:GFP. Amount of protein was 2.7 μg per lane. These plants were treated with either HS (42 °C for 1 h) or kept in control conditions. Immunoblotting analysis of FLOT2 with GFP tag was detected using anti‐GFP antibody. Loading controls of proteins on membrane were visualized using Novex reversible membrane protein stain. HS, heat stress; FLOT2, flotillin 2; GFP, green fluorescent protein; PM, plasma membrane. [file MPP-20-1005-s003.jpg]

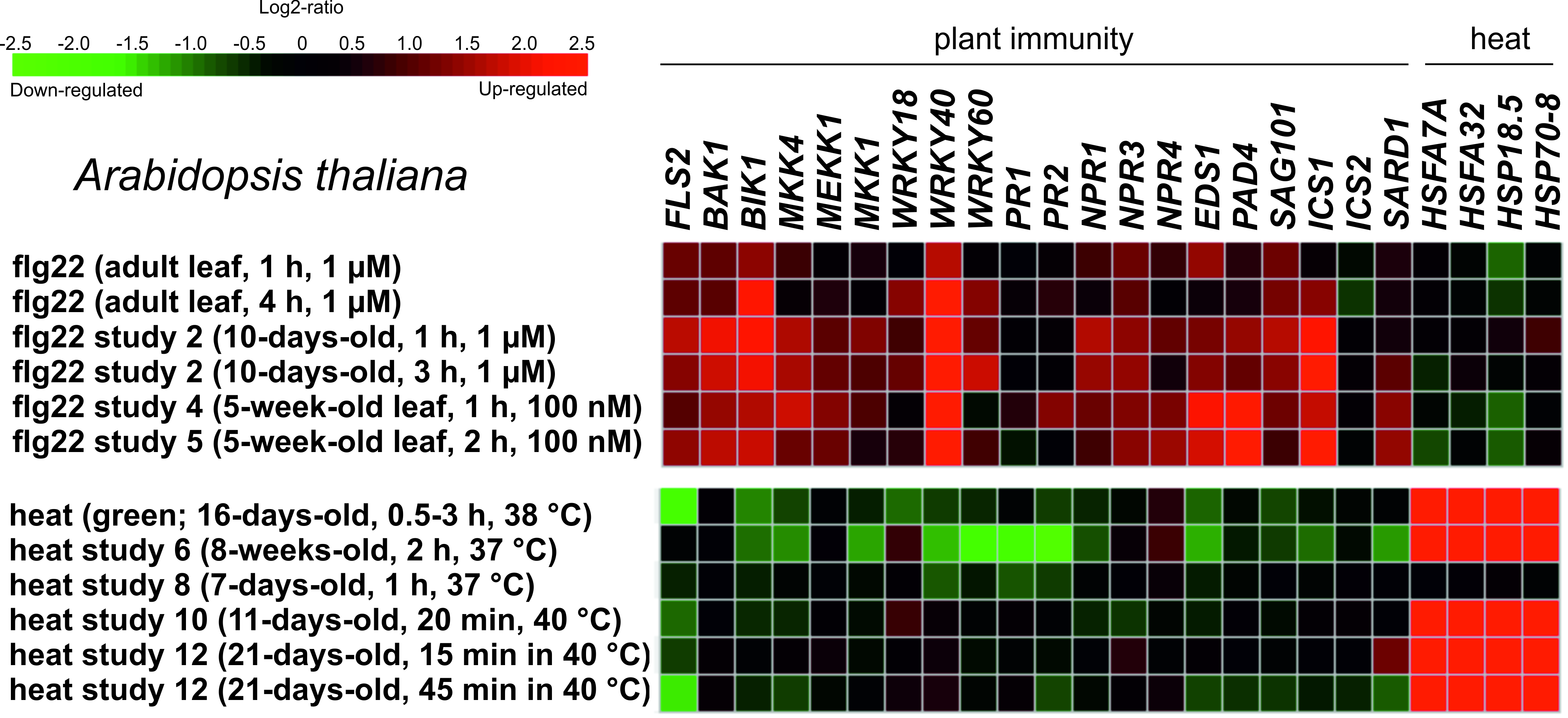

Supplement: Supplementary file 4 — Fig. S4 In silico analysis of the transcription of genes involved in plant immunity and HS. Transcription pattern of plant immunity response genes in A. thaliana. Diagrams show that selected genes responded differently to treatment by flg22 and HS. Experimental data were performed using Genevestigator (http://www.genevestigator.com). For list of genes see Table S1. HS, heat stress. [file MPP-20-1005-s004.jpg]

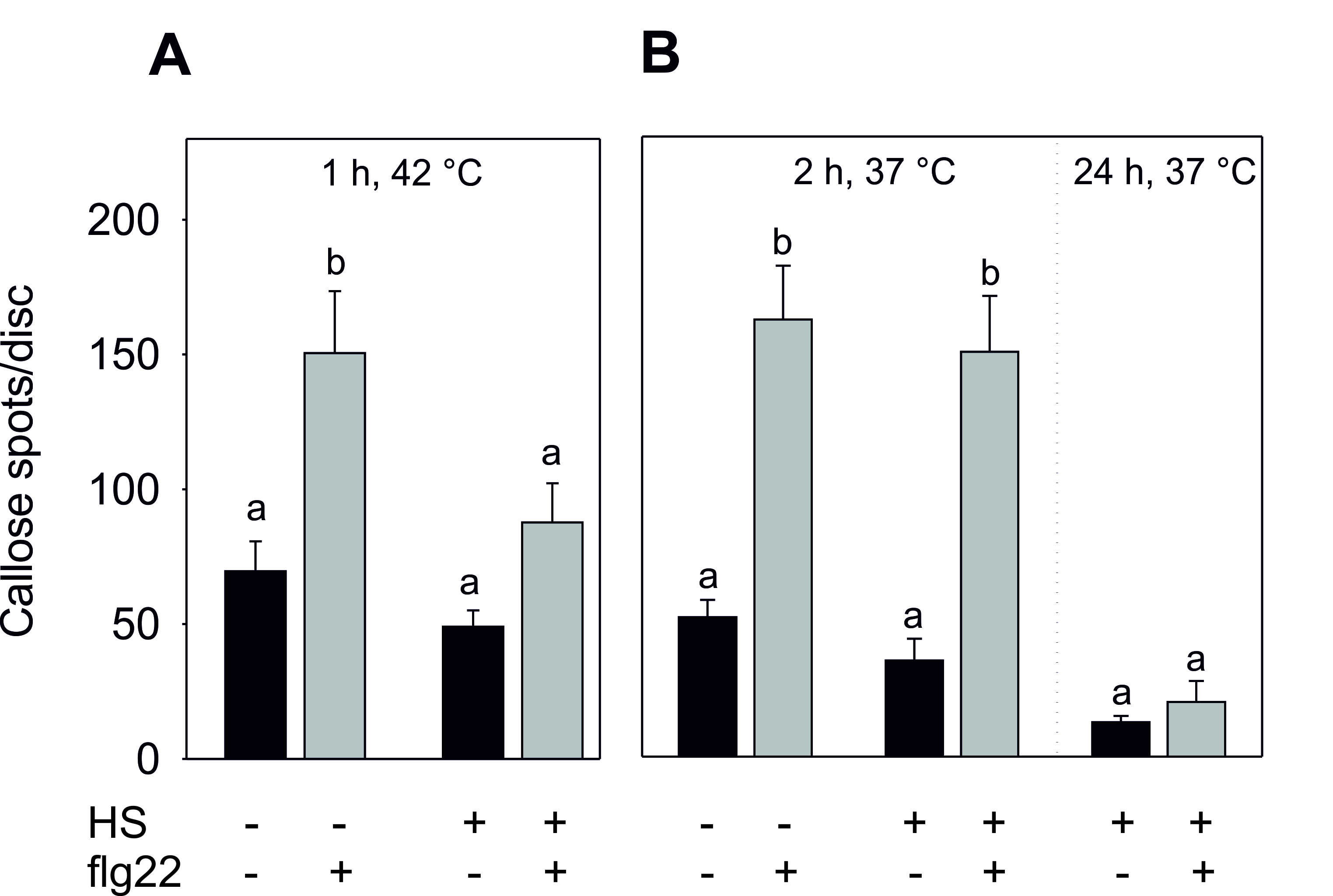

Supplement: Supplementary file 5 — Fig. S5 Callose deposition triggered by the combination of HS and flg22. Callose was stained with aniline blue and observed using fluorescent microscopy. The graph displays the amount of callose spots per 1 disc from 4‐week old A. thaliana plants treated with flg22 (100 nM, grey box) or without flg22 (black box) and with HS, A) 1 h at 42 °C and B) 2 h at 37 °C; 24 h at 37 °C. Treatment with flg22 took 24 h, and HS was applied either 1 h before the addition of flg22 (in the case of 1 h at 42 °C or 2 h at 37 °C treatment) or simultaneously with the flg22 treatment (in the case of 24 h at 37 °C). The data represent the means + SD; n = 30 leaf discs. One biological experiment was comprised of ten leaf discs. Values with different letters differed significantly at P < 0.05 based on a one‐way analysis of variance (ANOVA) with a post‐hoc Tukey HSD test. HS, heat stress, SD, standard deviation. [file MPP-20-1005-s005.jpg]

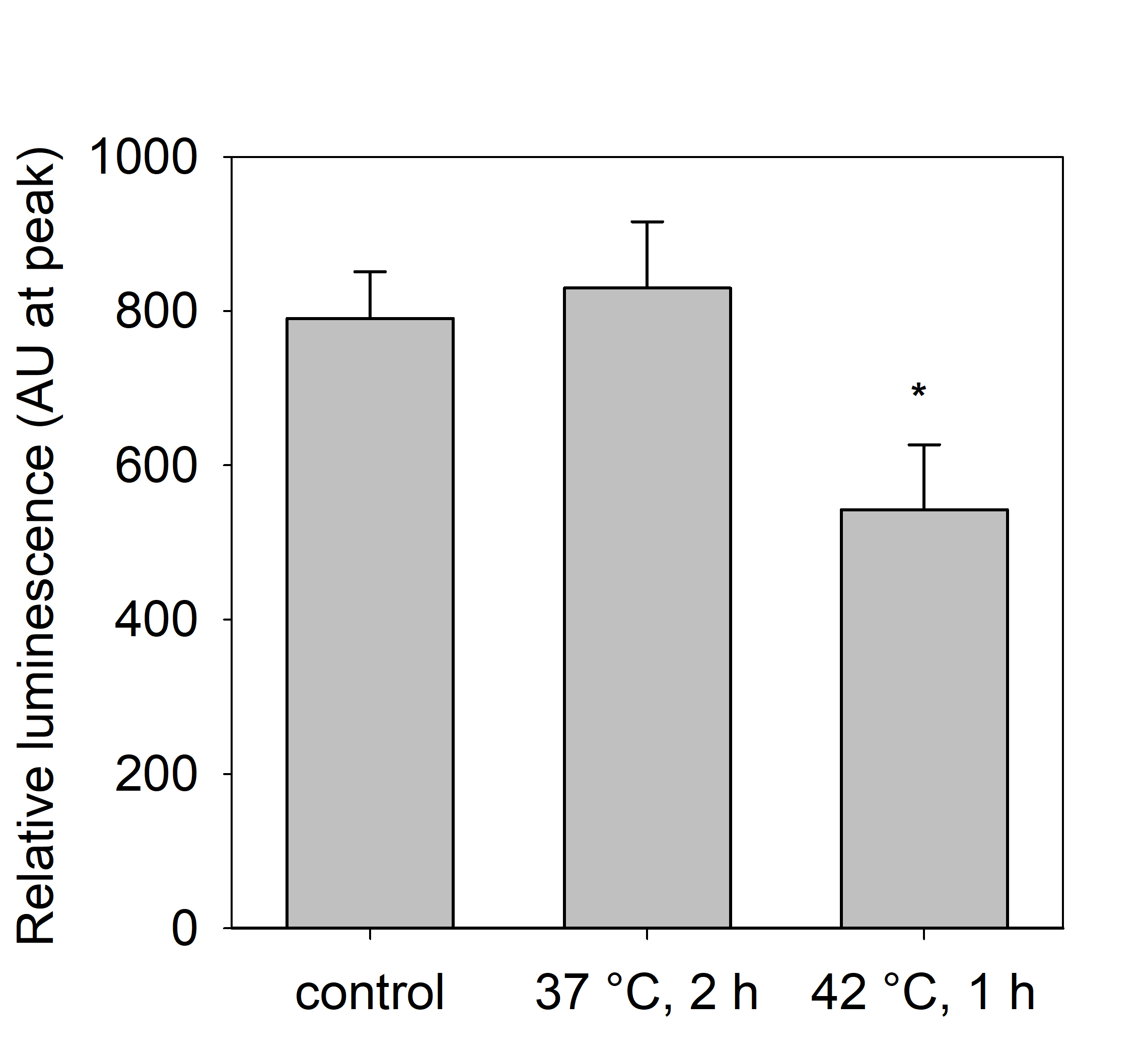

Supplement: Supplementary file 6 — Fig. S6 Oxidative burst triggered by flg22 after temporary HS. Four‐week‐old A. thaliana plants were exposed to 42 °C for 1 h and 37 °C for 2 h. After HS the plants were put back to the control conditions for 24 h. After that the leaf discs were cut and returned to 22 °C for 18 h. ROS production (represented by the peak luminescence) was measured after the addition of 100 nM flg22. The data represent means + SE; n = 12 discs. The experiment was repeated three times independently with similar results. The asterisks represent statistically significant changes between the heat and control conditions (*P < 0.5; two‐tailed Student´s t‐test). HS, heat stress; ROS, reactive oxygen species, SE, standard error. [file MPP-20-1005-s006.JPG]

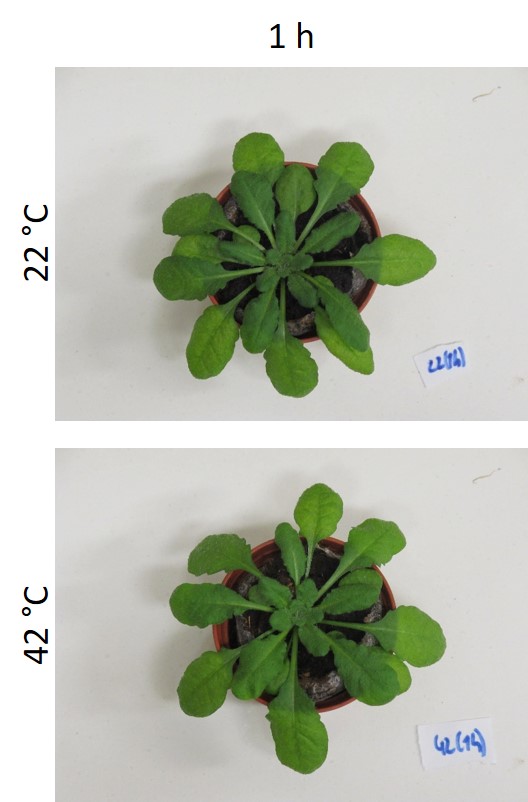

Supplement: Supplementary file 7 — Fig. S7 The phenotype of A. thaliana after heat stress (HS). Four‐week‐old A. thaliana plants were kept at 42 °C for 1 h and put back to the control conditions; 6 days after HS the pictures were taken. [file MPP-20-1005-s007.jpg]
