# Supplementary material for: Temporary heat stress suppresses PAMP‐triggered immunity and resistance to bacteria in Arabidopsis thaliana
Source: Mol Plant Pathol. 2019 Mar 29;20(7):1005–12. doi: 10.1111/mpp.12799 (PMC6589723; doi:10.1111/mpp.12799)
Supplement: Supplementary file 8 — Table S1 The phenotype of A. thaliana after heat stress (HS). Four‐week‐old A. thaliana plants were kept at 42 °C for 1 h and put back to the control conditions; 6 days after HS the pictures were taken. [file MPP-20-1005-s008.docx]

Table S1: List of genes selected for *in silico* gene transcription analysis after flg22 treatment and heat stress.

| *FLS2* | AT5G46330 | Flagellin-sensitive 2 |
| --- | --- | --- |
| *BAK1* | AT4G33430 | Brassinosteroid insensitive1-associated receptor kinase 1 |
| *BIK1* | AT2G39660 | Botrytis-induced kinase1 |
| *MKK4* | AT1G51660 | Mitogen-activated protein kinase kinase 4 |
| *MEKK1* | AT4G08500 | Mitogen-activated protein kinase kinase kinase 1 |
| *MKK1* | AT4G26070 | Mitogen-activated protein kinase kinase 1 |
| *WRKY18* | AT4G31800 | WRKY transcription factor 18 |
| *WRKY40* | AT1G80840 | WRKY transcription factor 40 |
| *WRKY60* | AT2G25000 | WRKY transcription factor 60 |
| *PR1* | AT2G14610 | Pathogenesis-related protein 1 |
| *PR2* | AT3G57260 | Glucan endo-1,3-beta-glucosidase |
| *NPR1* | AT1G64280 | Nonexpressor of PR genes 1 |
| *NPR3* | AT5G45110 | NPR1-like protein 3 |
| *NPR4* | AT4G19660 | NPR1-like protein 4 |
| *EDS1* | AT3G48090 | enhanced disease susceptibility 1 |
| *PAD4* | AT3G52430 | Phytoalexin deficient 4 |
| *SAG101* | AT5G14930 | Senescence-associated carboxylesterase 101 |
| *ICS1* | AT1G74710 | Isochorismate synthase 1 |
| *ICS2* | AT1G18870 | Isochorismate synthase 2 |
| *SARD1* | AT1G73805 | SAR Deficient 1 |
| *HSFA7A* | AT3G51910 | Heat stress transcription factor A-7a |
| *HSFA32* | AT4G21320 | Heat-stress-associated 32 |
| *HSP18.5* | AT2G19310 | 18.5 kDa class IV heat shock protein |
| *HSP70-8* | AT2G32120 | Heat shock 70 kDa protein 8 |
