# Supplementary material for: Temporary heat stress suppresses PAMP‐triggered immunity and resistance to bacteria in Arabidopsis thaliana
Source: Mol Plant Pathol. 2019 Mar 29;20(7):1005–12. doi: 10.1111/mpp.12799 (PMC6589723; doi:10.1111/mpp.12799)
Supplement: Supplementary file 9 — Table S2 Ion leakage 24 h after heat stress (HS) measured with a conductivity metre. [file MPP-20-1005-s009.docx]

Table S2: Ion leakage 24 h after HS measured with a conductivity meter.

|  |  | (%) |  |
| --- | --- | --- | --- |
| control | 2.014 | ± | 0.922 |
| HS (37 °C, 2 h) | 2.015 | ± | 0.341 |
| HS (42 °C, 1 h) | 2.029 | ± | 0.504 |

Each value is the mean ±SD (n=3). This experiment was repeated twice times with similar results.
