# Supplementary material for: Temporary heat stress suppresses PAMP‐triggered immunity and resistance to bacteria in Arabidopsis thaliana
Source: Mol Plant Pathol. 2019 Mar 29;20(7):1005–12. doi: 10.1111/mpp.12799 (PMC6589723; doi:10.1111/mpp.12799)
Supplement: Supplementary file 10 — Table S3 Sequence of specific primers. [file MPP-20-1005-s010.docx]

Table S3: Sequence of specific primers

| Locus | Name | Oligo Sequence (from 5´to 3´) | References |
| --- | --- | --- | --- |
| *At5g46330* | *FLS2* LP  *FLS2* PP | CTCCAGAGTTTGCTTATATGAG  TCCATATCAAGAACCCTAACC | This study |
| *At2g19190* | *FRK1* LP  *FRK1* PP | AATTAAGAGGCCAGATAGATCC  CCTTCAACGTTTAATTCGGTC | This study |
| *At1g74710* | *ICS1* LP  *ICS1* PP | GCAAGAATCATGTTCCTACC  AATTATCCTGCTGTTACGAG | Sasek et al., 2014 |
| *At4g34270* | *TIP41* LP  *TIP41* PP | GTGAAAACTGTTGGAGAGAAGCAA  TCAACTGGATACCCTTTCGCA | Czechowski et al., 2005 |

**Czechowski, T., Stitt, M., Altmann, T., Udvardi, M.K. and Scheible, W.R.** (2005) Genome-wide identification and testing of superior reference genes for transcript normalization in Arabidopsis. *Plant Physiology*, **139**,5-17.

**Sasek, V., Janda, M., Delage, E., Puyaubert, J., Guivarc'h, A., Maseda, E.L., Dobrev, P.I., Caius, J., Boka, K., Valentova, O., Burketova, L., Zachowski, A. and Ruelland, E.** (2014) Constitutive salicylic acid accumulation in pi4kIII beta 1 beta 2 Arabidopsis plants stunts rosette but not root growth. *New Phytologist*, **203**,805-816.
