# Supplementary material for: Temporary heat stress suppresses PAMP‐triggered immunity and resistance to bacteria in Arabidopsis thaliana
Source: Mol Plant Pathol. 2019 Mar 29;20(7):1005–12. doi: 10.1111/mpp.12799 (PMC6589723; doi:10.1111/mpp.12799)
Supplement: Supplementary file 11 — Methods S1 Experimental procedures. [file MPP-20-1005-s011.docx]

Methods S1: Experimental procedures

Plant growth conditions

In this study, *Arabidopsis thaliana* Columbia-0 (WT, Lehle seeds) and plants harbouring *pFLS::FLS2-GFP-HA* were used (Beck et al., 2012). Surface-sterilized seeds were sown on Jiffy 7 peat pellets. Plants were grown for 4 weeks under a short-day photoperiod (10 h/14 h light/dark cycle) at 100-130 μmol m^-2^ s^-1^, 22 °C and 70% relative humidity.

Measurement of ROS production

The ROS production was determined using the luminol-based assay as previously described (de Jonge et al., 2010; Krčková et al., 2018). The leaf discs of 4-week-old *A. thaliana* plants were either transferred to 28-42 °C for 15-360 min or kept in control conditions (22 °C, dark) and then put back to 22 °C. The discs were treated with 100 nM flg22 or 100 nM elf18 (EZbiolab) to induce the production of ROS.

Gene transcription analysis

The leaf discs of 4-week-old *A. thaliana* plants were either transferred to 42 °C for 15-45 min, to 37 °C for 120 min and to 28 °C for 360 min or maintained in control conditions (22 °C, dark). The samples were collected immediately after heat stress or transferred back to the control conditions for 2-8 h. The leaf discs from three independent wells were immediately frozen in liquid nitrogen. The tissue was homogenized in tubes with 1 g of 1.3 mm silica beads using a FastPrep-24 instrument (MP Biomedicals). Total RNA was isolated using a Spectrum Plant Total RNA kit (Sigma-Aldrich) and treated with a DNA-free kit (Ambion). Subsequently, 1 μg of RNA was converted into cDNA with M-MLV RNase H^–^ Point Mutant reverse transcriptase (Promega) and an anchored oligo dT21 primer (Metabion). Gene transcription was quantified by q-PCR using a LightCycler 480 SYBR Green I Master kit and LightCycler 480 (Roche). The PCR conditions were 95 °C for 10 min followed by 45 cycles of 95 °C for 10 s, 55 °C for 20 s, and 72 °C for 20 s. Melting curve analysis was then conducted. The relative transcription was normalized to the housekeeping gene *AtTIP41*. Primers were designed using PerlPrimer v1.1.21(Marshall, 2004). A list of the genes and primers analysed is available in Table S3.

Preparation of plasma membrane fraction and immunodetection of FLS2-GFP and FLOT2-GFP

The plasma membrane was purified from the leaves from 4-week-old *A. thaliana* plants harbouring pFLS::FLS2-GFP-HA or p35S::Flot2:GF-HA, which were treated either with heat stress (42 °C, 1 h) or kept in control conditions. Twelve plants and three fully developed leaves from each were used to partition microsomes in an aqueous dextran-polyethylene glycol two-phase system (Larsson et al., 1994; Novotná et al., 2003).

Briefly, homogenization buffer contained 50 mM HEPES-NaOH, pH 7.5, 0.4 mM sucrose, 0.1 M KCl, 0.1 M MgCl_2_, and protease inhibitors (0.23 mM PMSF, 0.83 mM benzamidine, 0.7 mM pepstatin, 1.1 mM leupeptin, and 77 nM aprotinin). The homogenate was filtered and centrifuged (6000 x g, 15 min, 4 °C), and the supernatant was centrifuged again (200 000 x g, 1 h, 4 °C). The pellet containing the microsomal membranes was resuspended in 5 mM phosphate buffer pH 7.8. Two grams of microsomal fraction were applied to the aqueous two-phase system (14 g) formed from dextran and polyethylene glycol 3350, both at a final concentration of 6.1% (w/w), 0.43 mM phosphate buffer pH 7.8, 3 mM KCl and 0.22 M sucrose. The plasma membrane fraction was diluted with 5 mM bistrispropane buffer, pH 6.0, and centrifuged (200 000 x g, 1 h, 4 °C). The purified plasma membrane pellet was resuspended in the 5 mM bistrispropane buffer pH 6.

The protein concentration of the plasma membrane fraction was determined using Coomassie plus protein assay reagent (Thermo Scientific). The Western blot analysis was performed according to the manufacturer's instructions. Proteins on gel were visualized using Imperial protein stain (Thermo Scientific) before WB and proteins on membrane were visualized using Novex reversible membrane protein stain (Invitrogen) after WB. The GFP tag was probed first with a mouse anti-GFP antibody (diluted 1:1000, Roche) and incubated with anti-mouse horseradish peroxidase conjugate (diluted 1:5000, Promega). The signals were developed using Super Signal West Pico Chemiluminescent Substrate (Thermo Scientific). Luminescence was detected on a G:box – Chemi 16 bio imaging system (Syngene).

Callose deposition

The leaf discs from 4-week-old *A. thaliana* plants were treated at 42 °C for 1 h and at 37 °C for 2 h before treatment with flg22 (1 µM for 24 h) or at 37 °C together with 1 µM flg22 (24 h). Distilled water was used as the control treatment. The discs were decolourized in ethanol:glacial acetic acid (3:1 v/v). The leaves were rehydrated in successive baths of 70% ethanol (at least 1 h), 50% ethanol (at least 1 h), 30% ethanol (at least 1 h) and water (at least 2 h). The discs were stained for at least 4 h with 0.01% aniline blue in 150 mM K_2_HPO_4_, pH 9.5. Callose deposition was observed using a Zeiss AxioImager ApoTome2 fluorescence microscope. Images were processed using Image/Fuji (Schindelin et al., 2012). At least 10 discs were analysed for each variant.

Bacterial inoculation

Bacterial *Pseudomonas syringae* pv *tomato* (*Pst*) strain DC3000 was used to inoculate 4‑week-old *A. thaliana* plants. *Pst* DC3000 was grown overnight on King B agar plates at 28 °C, resuspended in 10 mM MgCl_2_ and diluted to an OD_600_ of 0.001. Before inoculation, the plants were either exposed to high temperature (1 h at 42 °C, 2 and 5 h at 37 °C) or maintained in control conditions (22 °C). During this period, the plants were protected from drying in an incubator. After heat stress, the plants were uncovered and cultivated at 22 °C for an additional 18 h. All the plants were kept in the dark during the whole period. Using a needleless syringe, the bacterial suspension was infiltrated into three fully developed leaves from one plant. After 3 days, leaf discs (6 mm in diameter) from the infected leaves were collected (3 discs from one plant represent one sample). The leaf discs were homogenized in tubes with 1 g of 1.3 mm silica beads using a FastPrep-24 instrument (MP Biomedicals, USA). The resulting homogenate was serially diluted and pipetted onto King B plates. The colonies were counted after 1-2 days of incubation at 28 °C.

Ion leakage

The 4-week-old *A. thaliana* plants were exposured to HS (2 h at 37 °C or 1 h at 42 °C) and returned to the control conditions (22 °C) for 24 h. Ion leakage was estimated according to Prerostova (2018). Briefly, five leaves were incubated for 24 h in double-distilled water at 4 °C in the dark. The electrical conductivity (C1) was measured with COND70 conductivity meter (XS Instruments). The samples were boiled in a water bath for 20 min, then the electrical conductivity was measured again (C2). Ion leakage was calculated as (C1/C2) x 100 [%].

**Beck, M., Zhou, J., Faulkner, C., MacLean, D. and Robatzek, S.** (2012) Spatio-temporal cellular dynamics of the *Arabidopsis* flagellin receptor reveal activation status-dependent endosomal sorting. *The Plant Cell*, **24**,4205-4219.

**de Jonge, R., van Esse, H.P., Kombrink, A., Shinya, T., Desaki, Y., Bours, R., van der Krol, S., Shibuya, N., Joosten, M.H. and Thomma, B.P.** (2010) Conserved fungal LysM effector Ecp6 prevents chitin-triggered immunity in plants. *Science*, **329**,953-955.

**Krčková, Z., Kocourková, D., Daněk, M., Brouzdová, J., Pejchar, P., Janda, M., Pokotylo, I., Ott, P.G., Valentová, O. and Martinec, J.** (2018) The *Arabidopsis thaliana* non-specific phospholipase C2 is involved in the response to *Pseudomonas syringae* attack. *Annals of Botany*, **121**,297-310.

**Larsson, C., Sommarin, M. and Widell, S.** (1994) Isolation of highly purified plant plasma-membranes and separation of inside-out and right-side-out vesicles. *Aqueous Two-Phase Systems*, **228**,451-469.

**Marshall, O.J.** (2004) PerlPrimer: cross-platform, graphical primer design for standard, bisulphite and real-time PCR. *Bioinformatics*, **20**,2471-2472.

**Novotná, Z., Martinec, J., Profotová, B., Žďárová, S., Kader, J.C. and Valentová, O.** (2003) In vitro distribution and characterization of membrane-associated PLD and PI-PLC in *Brassica napus*. *Journal of Experimental Botany*, **54**,691-698.

**Prerostova, S., Kramna, B., Dobrev, P.I., Gaudinova, A., Marsik, P., Fiala, R., Knirsch, V., Vanek, T., Kuresova, G. and Vankova, R.** (2018) Organ-specific hormonal cross-talk in phosphate deficiency. *Environmental and Experimental Botany*, **153**,198-208.

**Schindelin, J., Arganda-Carreras, I., Frise, E., Kaynig, V., Longair, M., Pietzsch, T., Preibisch, S., Rueden, C., Saalfeld, S., Schmid, B., Tinevez, J.-Y., White, D.J., Hartenstein, V., Eliceiri, K., Tomancak, P. and Cardona, A.** (2012) Fiji: an open-source platform for biological-image analysis. *Nature Methods*, **9**,676.
